# Atención inicial de migrantes en Chile: iniciativa en atención primaria de salud a un año de su implementación

**DOI:** 10.26633/RPSP.2019.71

**Published:** 2019-09-06

**Authors:** Macarena Chepo, Sofía Astorga-Pinto, Báltica Cabieses

**Affiliations:** 1 Programa de Estudios Sociales en Salud Facultad de Medicina Clínica Alemana Universidad del Desarrollo Santiago Chile Programa de Estudios Sociales en Salud, Facultad de Medicina Clínica Alemana, Universidad del Desarrollo, Santiago, Chile.

**Keywords:** Migrantes, sistemas de salud, accesibilidad a los servicios de salud, atención primaria, Chile., Transients and migrants, health systems, health services accessibility, primary health care, Chile, Migrantes, sistemas de saúde, acesso aos serviços de saúde, atenção primária à saúde, Chile.

## Abstract

**Objetivo.:**

Describir las características sociodemográficas, necesidades de salud, derivaciones efectivas realizadas y evaluación inmediata de la población migrante internacional que participó en el Programa de Atención Inicial al Migrante durante el primer año de ejecución (entre mayo y diciembre del año 2016), implementado en el Centro de Salud Familiar Ignacio Domeyko, Santiago de Chile.

**Métodos.:**

Estudio descriptivo. Se diseñó e implementó una intervención para dar bienvenida a migrantes internacionales, que contemplaba la evaluación integral inicial, el diagnóstico de situación y detección de necesidades y derivación a otras atenciones, y la entrega de información. Para el análisis se describen, por medio de medidas de tendencia central y frecuencias absolutas y relativas, las características sociodemográficas, el proceso migratorio, las necesidades de salud, el cumplimento de derivaciones a un año de seguimiento y los resultados de la encuesta de satisfacción usuaria.

**Resultados.:**

Se inscribieron 436 personas, de las cuales asistieron 270 (61,9%). El 80% eran mujeres, provenientes en su mayoría de Perú y Venezuela. La principal derivación realizada fue a control de embarazo (32,6%), seguido de planificación familiar (30%) y servicios sociales (27,04%). A un año de seguimiento, las derivaciones para controles cardiovasculares alcanzaron 100% de cumplimiento, 97,7% para embarazo y 87,7% para servicios sociales. El menor cumplimiento en las derivaciones fue a salud mental (11,1%).

**Conclusiones.:**

Esta intervención, pionera en Chile, permitió dar bienvenida y proporcionar información clave a la población migrante internacional, así como también realizar derivaciones basadas en necesidades de salud y promover la inserción de la población inmigrante al sistema de salud chileno.

## Abstract (en)

**Objective.:**

Describe sociodemographic characteristics, health needs, effective referrals, and immediate evaluation of international migrants who participated in the Initial Care for Migrants program during its first year of implementation (May to December 2016) at the Ignacio Domeyko Family Health Center in Santiago, Chile.

**Methods.:**

Descriptive study. An intervention was designed and implemented to welcome international migrants. It included an initial comprehensive evaluation, a situation assessment, identification of needs, referral to other health services, and transfer of information. Statistical analysis was performed, using measures of central tendency and absolute and relative frequencies to describe sociodemographic characteristics, migratory processes, health needs, and effective referrals after one year of monitoring, as well as the results of a user satisfaction survey.

**Results.:**

436 people were registered, of whom 270 (61.9%) participated; 80% were women, mostly from Peru and Venezuela. The largest number of referrals were to pregnancy services (32.6%), followed by family planning (30%) and social services (27.04%). After one year of monitoring, there was 100% compliance for referrals to cardiovascular testing, 97.7% for pregnancy, and 87.7% for social services. The lowest compliance was for referrals to mental health services (11.1%).

**Conclusions.:**

This intervention, the first of its kind in Chile, welcomed international migrants and provided them with key information, while providing referrals based on health needs and promoting the inclusion of the immigrant population in the Chilean health system.

## RESUMO

**Objetivo.:**

Descrever as características sociodemográficas, necessidades em saúde, encaminhamentos efetivados e avaliação imediata da população migrante internacional que participou do Programa de Atenção Inicial ao Migrante no primeiro ano da sua implementação (entre maio e dezembro de 2016) no Centro de Saúde Familiar Ignacio Domeyko, na cidade de Santiago, no Chile.

**Métodos.:**

Estudo descritivo com base em uma intervenção que foi concebida e implementada para acolher populações migrantes internacionais. A intervenção consistiu de uma avaliação inicial completa, diagnóstico da situação, identificação das necessidades em saúde e encaminhamento a outros serviços de atendimento, além de um componente informativo. A análise se baseou em medidas de tendência central e frequências absolutas e relativas usadas para descrever características sociodemográficas, processo migratório, necessidades em saúde, efetivação dos encaminhamentos em um ano de acompanhamento e resultados da pesquisa de satisfação dos usuários.

**Resultados.:**

Dos 436 inscritos, 270 participaram do programa (61,9%). Destes, 80% eram mulheres e a maioria era proveniente do Peru e da Venezuela. Os principais encaminhamentos efetivados foram ao atendimento pré-natal (32,6%), planejamento familiar (30%) e serviços sociais (27,04%). Após um ano de acompanhamento, os encaminhamentos para avaliação cardiovascular foram efetivados em 100%, para atenção pré-natal em 97,7% e para serviços sociais em 87,7%. O encaminhamento à atenção de saúde mental foi o menos efetivado (11,1%).

**Conclusões.:**

Esta foi uma intervenção pioneira no Chile que possibilitou acolher e instruir as populações migrantes internacionais, além de realizar encaminhamentos a serviços conforme as necessidades em saúde e promover a inserção da população imigrante no sistema de saúde chileno.

La migración internacional es un fenómeno global y complejo, que en la actualidad ha alcanzado cifras sin precedentes. Se estima que en 2017 existían 258 millones de migrantes internacionales (3,3% de la población mundial) (1). En años recientes, se registró un aumento de 15% de los flujos migratorios hacia países de la Organización para la Cooperación y el Desarrollo Económicos (OCDE), entre los que Chile destaca con un incremento de 33% en las nuevas entradas registradas entre 2015 y 2016 (2). En la actualidad, se estima que hay en el país un total de 1 251 225 migrantes internacionales (7,1% de la población total) (3). El flujo migratorio predominante es sursur, distribuido en países como Venezuela (23%), Perú (17,9%), Haití (14,3%) y Colombia (11,7%) (3). Un 5,6% del total de la población migrante en Chile (7000 personas) correspondería a refugiados y solicitantes de asilo (4).

Existe consenso en afirmar que la migración internacional es un determinante social de la salud, ya que la movilidad humana a escala internacional impacta de manera significativa en la disposición de recursos en salud y planificación sanitaria, así como también en los resultados de salud del país que se deja atrás y del país al cual se llega (5). Se ha descrito que la población migrante enfrenta importantes barreras de acceso a servicios de salud, las cuales operan en distintos niveles: estructural (barreras de tipo cultural y político), institucional (barreras de tipo organizacional y de prestación de servicios) y a nivel individual (características propias de la población migrante internacional y de los proveedores de servicios) (6, 7). La literatura internacional disponible reconoce que estas barreras influyen de forma negativa en los resultados de salud de la población migrante y, además, genera diferencias injustas y evitables entre la población migrante internacional y la población local a lo largo del tiempo (8).

En Chile, la migración internacional ha recibido especial atención en materia legislativa y de salud en el último período. En época reciente, se ha promulgado la Política de Salud del Migrante Internacional, iniciativa que promueve una mirada inclusiva de la migración, en particular desde el enfoque de los derechos humanos (9). Nuestro país ha garantizado que los extranjeros que cuenten con un permiso vigente para permanecer en el país tienen derecho al acceso a la salud en igualdad de condiciones que los ciudadanos chilenos. Asimismo, personas en situación de emergencias, control prenatal y menores de 18 años, cualquier sea su situación migratoria, pueden acceder a prestaciones de salud brindadas por el sistema público. Se incluye en este grupo también a personas en situación migratoria irregular que acrediten encontrarse en situación de carencia de recursos, ante lo cual pueden optar por inscribirse en el sistema público con un código provisorio mientras reciben su visa temporaria (9).

No obstante estos esfuerzos por integrar al sistema de salud a migrantes internacionales en Chile, según estimaciones calculadas por las autoras en base a la Encuesta CASEN (10), existen importantes diferencias en el acceso y uso de servicios de salud entre chilenos e inmigrantes. Para el año 2017, 15,8% de la población adulta migrante internacional declaró no contar con ningún tipo de previsión de salud (en comparación con 2,2% de la población local). Según el sexo, las mujeres inmigrantes presentan un mayor porcentaje de afiliación al sistema público de salud, con 68,4% del total (en los hombres es de 61,7%).

Desde la 61.ª Asamblea Mundial de la Salud en 2008 (11), la Organización Mundial de la Salud (OMS) ha realizado diferentes llamados orientados al fomento del acceso equitativo de salud de la población migrante, sin discriminación por motivos de género, religión, nacionalidad o etnia. En esta línea, y como una forma de mejorar el acceso libre e igualitario al sistema de salud público de la población inmigrante que reside en Chile, hace tres años se implementó una estrategia de bienvenida al sistema de salud chileno para atención primaria (APS) denominada Programa de Atención Inicial a Migrantes. Esta iniciativa basada en ciencia fue desarrollada en el marco de un trabajo colaborativo con la Dirección de Salud Municipal de la Comuna de Santiago, Región Metropolitana (RM). A la fecha, y luego de tres años de ejecución de este programa, se han realizado cerca de 1800 atenciones de salud, lo que representa 1,6% de la población residente en la comuna (12).

El objetivo del presente artículo es describir las características sociodemográficas, necesidades de salud, derivaciones efectivas realizadas y evaluación inmediata de parte de la población migrante internacional que participó en el Programa de Atención Inicial a Migrantes durante el primer año de ejecución (de mayo a diciembre del año 2016) y que fue implementado en el Centro de Salud Familiar (CESFAM) Ignacio Domeyko, Santiago de Chile, (RM).

## MATERIALES Y MÉTODOS

Se trata de un estudio observacional, de tipo descriptivo, basado en datos provenientes del registro de las atenciones otorgadas en el Programa de Atención Inicial implementado en el CESFAM Ignacio Domeyko, Comuna de Santiago, RM.

Se incluyó a la totalidad de la población migrante inscrita en el Programa de Atención Inicial a Migrantes entre mayo y diciembre del año 2016 (n = 436). Según datos del CENSO 2017, en la RM habita la mayoría de la población migrante del país (62,8%). De las 52 comunas que tiene la RM, Santiago concentra el mayor porcentaje de población migrante internacional (23,6%) (12). Dentro de la comuna, el CESFAM Ignacio Domeyko tiene la mayor proporción de población extranjera (22,4%, con un total de 12 810 inscritos) (13).

**FIGURA 1:**
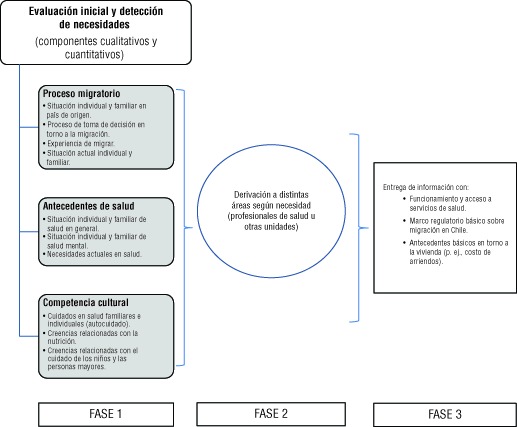
Modelo de Atención del Programa de Atención Inicial a Migrantes, Santiago, Chile

El programa incorporó un modelo de atención de tres fases (figura 1):

1. Fase 1. Evaluación integral inicial de la persona o grupo familiar: en esta fase se realiza un diagnóstico de la situación del migrante y su grupo familiar, en especial sobre necesidades concretas y urgentes en salud (también otros aspectos relevantes para el proceso de inserción en el país, como necesidades de educación de los hijos y trámites para la visa, entre otros). Se incluyeron preguntas sobre el proceso migratorio, historia de salud y competencias interculturales. Sobre este último concepto, el objetivo de su consideración en este protocolo de atención fue promover, en trabajadores de la salud, conocimientos, actitudes y habilidades distintivas para el desempeño con personas de otras culturas (14). Para ello, se realizaron preguntas dirigidas a creencias con respecto a la nutrición y cuidados de salud en general para sí mismos y su familia.
2. Fase 2. Derivaciones a otras atenciones según necesidad: a partir de las necesidades detectadas en la fase 1, los participantes fueron derivados o referidos a otros servicios dentro del mismo centro de salud, los cuales pueden incluir más de una derivación según cada necesidad particular (por ejemplo, exámenes preventivos, control de salud con un profesional de salud o servicios sociales, entre otros).
3. Fase 3. Entrega de información esencial: al finalizar la entrevista, se entregaba información básica con respecto al funcionamiento y organización del sistema de salud chileno, derechos en salud y localización geográfica de otros centros de salud de la red de atención (por ejemplo, servicio de urgencia), entre otros. También se entregó información general sobre el acceso al sistema educacional y el marco regulatorio básico en torno a la migración, como apoyo adicional para la integración social efectiva.

Para la evaluación inicial, se elaboró un cuestionario de atención que contenía diversas preguntas de tipo demográficas, sobre la experiencia migratoria y preguntas de “bandera roja”. Estas últimas eran una señal de alerta ante situaciones de vulnerabilidad social urgente que requiriesen derivación inmediata a algún dispositivo de atención (como asistencia social, consulta de urgencia y evaluación médica, entre otros). Las preguntas del protocolo de atención de este programa (15) se elaboraron con base en lo documentado en diversos estudios previos realizados por el equipo de investigación (16–21). El detalle de este protocolo de atención puede ser solicitado a la autora de correspondencia de este manuscrito.

Antes del inicio del programa, un equipo de profesionales de salud (matronas, médicos, enfermeras y psicólogas) recibieron entrenamiento sobre el modelo y la aplicación del cuestionario. Se programaron las consultas con una duración de 45 minutos, en el horario de 17:00 a 20:00 horas, dos veces por semana. Todos los profesionales entrenados aplicaron el cuestionario. Las autoridades del centro de salud difundieron el programa de atención hacia los trabajadores de salud. Las personas atendidas debían tener, como máximo, un año de residencia en Chile y podían ser derivados desde atenciones con otros profesionales o desde el mesón de inscripción al centro de salud o por consulta espontánea.

## Variables del estudio

1. Características sociodemográficas: país de origen, sexo, edad, condición de hacinamiento (calculado a partir de la cantidad de personas por dormitorio, clasificadas en “sin hacinamiento” cuando existen 2,4 personas o menos por dormitorio; “hacinamiento medio” con 2,5 a 4,9 personas por dormitorio y “hacinamiento crítico” cuando hay más de cinco personas por dormitorio), tipo de vivienda (residencia actual, que puede ser casa, departamento o dormitorio, propio o en arriendo, o también “otro”, en caso de allegamiento o residencias temporales), ingresos (cantidad de ingresos totales de la vivienda, medidos en pesos chilenos y convertidos a dólares estadounidenses [USD] al cambio vigente al 7 junio de 2019).
2. Características del proceso migratorio: calificación personal respecto de la experiencia migratoria (escala del 1 al 7, siendo 1 la peor experiencia y 7 como excelente experiencia), situación migratoria (situación migratoria vigente en Chile al 2016, según el tipo de visa) y proporción de remesas (proporción del ingreso que es enviada al país de origen).
3. Características de la atención de salud: motivo de consulta (referido por la persona migrante), cantidad y tipo de derivaciones realizadas (profesional o tipo de atención o atenciones a las que fue derivada la persona migrante, en caso de ser necesario) y demanda satisfecha (cantidad de derivaciones efectivamente cumplidas a un año de seguimiento). Este último indicador fue entendido como “cumplido” cuando existía registro en la ficha clínica de una atención de salud concretada con el profesional correspondiente a la derivación realizada, a un año de elevada la solicitud.

Al final de la atención entregada, se les pidió a los participantes responder un breve cuestionario de autorrespuesta de satisfacción usuaria, el cual contenía una escala tipo Likert que consideraba las categorías: “muy satisfecho”, “satisfecho”, “poco satisfecho” y “nada satisfecho”. Los ítems evaluados fueron: espacio físico, tiempo dedicado, comunicación y lenguaje del profesional, preguntas realizadas, trato del profesional y limpieza y higiene del lugar. Además, se agregó una pregunta dicotómica (sí/no) respecto a si la persona consideraba haber obtenido la atención que esperaba.

## Análisis estadístico

Los datos de la población atendida se obtuvieron de las fichas de atención (preguntas cuantitativas), codificados y vertidos en una base de datos en Excel®. El análisis estadístico descriptivo se realizó con medidas de tendencia central, frecuencias absolutas y relativas.

## Aspectos éticos

Este proyecto fue aprobado por las autoridades de salud de la Ilustre Municipalidad de Santiago. Cada una de las personas fue invitada a participar en el estudio de manera voluntaria vía firma de consentimiento informado, documento que explicitaba la confidencialidad en el manejo de información obtenida y de los datos personales. Esta situación es especialmente relevante debido a que cada persona entregaba información respecto a su situación migratoria. Asimismo, el diseño de este programa de atención inicial se desarrolló en el marco del Proyecto de Investigación Fondecyt 11130042, el cual contó con la aprobación del Comité de Ética de la Universidad del Desarrollo y del Comité de Ética de Fondecyt, Comisión Nacional de Investigación Ciencia y Tecnología, Gobierno de Chile.

## RESULTADOS

## Características sociodemográficas

**FIGURA 2:**
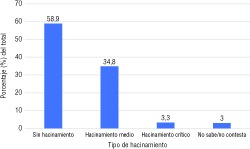
Situación de hacinamiento en la población perteneciente al Programa de Atención Inicial al Migrante (mayo a diciembre 2016)

Desde mayo a diciembre se 2016 se inscribieron 436 personas, de las cuales asistieron un total de 270 (61,9%). Se revisaron la totalidad de las fichas y registros de este grupo, cuyas características sociodemográficas se describen en el cuadro 1. La edad promedio fue 30,4 años (DE 9,08), la mayoría fueron mujeres (83%) provenientes de Venezuela (41,1%, n = 111), Perú (24,8%, n = 67) y Haití (10,7%, n = 29). Respecto al tipo de vivienda, la mayoría se encontraba en situación de arriendo, ya sea de casa o departamento (50%, n = 135) o de dormitorio (41,1%, n = 111). El ingreso promedio mensual por hogar referido por las personas migrantes fue de USD 397,8 (rango USD 0-2167). Con relación al hacinamiento (figura 2), 34,8% (n = 94) de los participantes vivía en situación de hacinamiento medio y 3,3% (n = 9) vivía en hacinamiento crítico.

**CUADRO 1.** Características sociodemográficas y situación migratoria de la población en estudio perteneciente al Programa de Atención Inicial al Migrante (mayo a diciembre de 2016)

Variable	Muestra	
	N = 270	%
Edad (años)	Promedio: 30,4	
	DE: 9,1	
	Rango: 17-69	
Sexo		
Femenino	224	83,0
Masculino	46	17,0
País de origen		
Argentina	2	0,7
Bolivia	4	1,5
Brasil	1	0,4
Colombia	37	13,7
Ecuador	8	3,0
Haití	29	10,7
Perú	67	24,8
República Dominicana	8	3,0
Venezuela	111	41,1
México	1	0,4
No sabe/no contesta	2	0,7
Ingresos	Promedio: USD 397,8^a^	
	Rango: USD 0-2167^a^	
Tipo de vivienda		
Casa o departamento en arriendo	135	50,0
Dormitorio en arriendo	111	41,1
Vivienda propia	4	1,5
Otra	18	6,7
No sabe/no contesta	2	0,7
Situación migratoria		
Visa en trámite	145	53,7
Visa sujeta a contrato	15	5,6
Visa turista	71	26,3
Visa temporaria	4	1,5
Situación irregular	31	11,5
Situación regular	2	0,7
No sabe/no contesta	2	0,7
Nota experiencia migratoria		
1	6	2,2
2	1	0,4
3	4	1,5
4	21	7,8
5	59	21,9
6	67	24,8
7	79	29,3
No sabe/no contesta	33	12,2
Envío de remesas al país de origen		
Sí	58	21,5
No	212	78,5
Proporción de remesas enviadas respecto del ingreso (%)	Promedio: 25,7	
	DE: 14,9	
	Rango: 3,3-62,5	

USD; dólares estadounidenses.

a Valor al cambio vigente el 7 de junio de 2019: 1 USD = 692,2 pesos chilenos.

Cuadro de elaboración propia.

**FIGURA 3:**
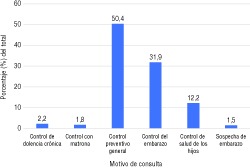
Principales motivos de consulta referidos por la población en estudio perteneciente al Programa de Atención Inicial al Migrante (mayo a diciembre de 2016)

## Características del proceso migratorio

La mayoría de las personas señaló como situación migratoria de “visa en trámite” (53,7%, n = 145), seguido de “visa de turista” (26,3%, n = 71) y “en situación irregular” (11,5%, n = 31). La calificación de la experiencia migratoria se distribuyó sobre todo entre las notas 5 y 7, con esta última como la registrada con mayor frecuencia (29,26%, n = 79). Un 21,5% de las personas indicó haber enviado remesas a su país de origen (n = 58), monto en dinero que representó entre 3,3% y 62,5% del ingreso total del hogar (cuadro 1).

## Características de la atención de salud

El principal motivo de consulta (figura 3) referido fue el control preventivo (50,4%, n = 136), seguido por el control de embarazo (31,9%, n = 86) y para control de salud preventivo de sus hijos (12,2%, n = 33). Respecto a las derivaciones a otros profesionales o unidades disponibles en el centro de salud (cuadro 2), la más frecuente fue la derivación al control de embarazo (32,6%), seguida por la derivación a matrona (30%) y a servicios sociales (27,04%). A un año de seguimiento, el cumplimiento de estas derivaciones fue de 100% para los controles cardiovasculares, seguido por el control de embarazo (97,7%), el de servicios sociales (87,7%) y el control de salud preventivo para los hijos (82,5%). El menor cumplimiento fue a salud mental, donde solo un 11,1% recibió atención por un profesional del área.

Respecto a los resultados de satisfacción usuaria, del total de asistentes se recolectó información de 146 personas (54% del total de atenciones efectivas realizadas). De estas, la totalidad se manifestó como “muy satisfecho” y “satisfecho”. Un 73,6% de los participantes indicó estar “muy satisfechos” con el espacio físico, el tiempo dedicado a la atención (82,19%), la comunicación y el lenguaje profesional (92,47%), las preguntas que se realizaron (80,82%), el trato del profesional (95,2%) y la limpieza e higiene del lugar (81,51%). Por último, se les preguntó si recibieron la atención que esperaban y 95,58% de las personas respondieron “sí”.

**CUADRO 2.** Distribución de derivaciones realizadas según profesionales o unidades de atención y proporción de cumplimiento de dichas referencias

Profesional o tipo de atención a quien fue derivado	N^a^	Porcentaje del total de referencias (%)^b^	Cumplimiento (%)^c^
Control de embarazo	88	22,7	97,7
Médico	30	7,8	76,7
Salud mental	9	2,3	11,1
Servicios sociales	73	19,9	87,7
Control cardiovascular	6	1,6	100,0
Control de salud preventivo de hijos	40	10,3	82,5
Matrona	81	20,9	69,1
Servicio de urgencia	6	1,6	50,0
Examen preventivo del adulto (EMPA)	54	13,9	59,3
TOTAL	387	100,0	

Cuadro de elaboración propia.

a Cantidad de derivaciones realizadas según el profesional o la unidad.

b Proporción del total de derivaciones realizadas a la población en estudio.

c Proporción de las derivaciones efectivamente cumplidas; esto es, en las que existió registro posterior de la atención de salud con el profesional o unidad a la cual fue derivada la persona.

## DISCUSIÓN

El Programa de Atención Inicial a Migrantes es una de las primeras estrategias exitosas e innovadoras documentadas a la fecha en APS. Cuenta con una evaluación formal del primer año de ejecución que se describe en este artículo y que permite promover e inspirar iniciativas similares para el resto de la región de América Latina y el Caribe. Este programa tuvo como objetivo general dar la bienvenida al sistema de salud público a la población migrante internacional por medio de un protocolo estandarizado y simple, que fue diseñado a partir de la evidencia científica nacional disponible a la fecha. Esta iniciativa representa un ejemplo concreto de traslación del conocimiento de investigación a la acción en salud pública para la inclusión de migrantes internacionales al país de acogida, con un enfoque basado en ciencia y en el respeto a los derechos humanos (22).

Con relación a los resultados obtenidos, solo 61,9% de las personas concretaron su atención. La literatura internacional describe múltiples barreras de acceso que podrían explicar la alta inasistencia observada, se destacan las barreras idiomáticas, el miedo a ser deportados y no saber cómo navegar en el sistema, entre otros (6, 23, 24). Otro factor que puede ser explicativo es que el programa de atención inicial no contaba (durante el período de estudio) con un mecanismo de confirmación o recordatorio de citas. En la actualidad, hay diversas estrategias para mejorar la asistencia al programa, como el desarrollo de dispositivos de confirmación telefónica y la incorporación de un facilitador lingüístico de origen haitiano, cuya función es favorecer el diálogo entre el profesional y la persona migrante en caso de no hablar español. Por último, también se han programado reuniones informativas periódicas con los trabajadores de este y otros centros de salud que han implementado el programa, con la intención de difundir el propósito, las actividades y los logros de esta iniciativa.

La mayoría de quienes asistieron son mujeres. Los motivos de consulta más frecuentes fueron el control preventivo (50,4%) y el control del embarazo (31,9%). En Chile, las mujeres migrantes reportan un mayor uso de servicios de salud comparado con hombres (25). Una de las razones que podrían explicar esta diferencia es la mayor disponibilidad de servicios de salud preventivos para mujeres en el país, sobre todo en materia de salud sexual y reproductiva (9). Cabe destacar que la gran proporción de participación femenina en este programa releva la necesidad de incorporar una perspectiva de género, con la finalidad de comprender cómo este factor intersecta con otros determinantes de la salud como la etnia, la estratificación social y la discriminación, entre otros.

Una elevada proporción de la población asistente a este programa se encontraba en situación de hacinamiento. Estos datos concuerdan con los resultados a nivel nacional, donde para el año 2017 las personas migrantes en condiciones de hacinamiento crítico alcanzan 3,9% frente a 0,8% alcanzado por la población local. Por otra parte, estudios disponibles demuestran las variadas dificultades en vivienda y habitabilidad que enfrenta este grupo, sumadas a los riesgos que esta situación conlleva para la salud y el bienestar de estas familias (16, 25). Para el caso de la comuna de Santiago, una elevada proporción de la población migrante reside en espacios reducidos, con escasas medidas de seguridad y, muchas veces, en condiciones de subarrendamiento a elevado costo (25). Todos estos factores deben ser indagados en el contexto de la atención clínica, considerando la vulnerabilidad social y de salud que revisten para las personas migrantes y sus familias.

Una escasa proporción de la población estudiada fue referida a unidades de salud mental. Una posible explicación a este fenómeno, bajo un enfoque de salud intercultural, son las diversas conceptualizaciones de salud y bienestar de cada individuo (27). En este sentido, las personas migrantes internacionales pueden no considerar necesario consultar frente a ciertas condiciones de salud. Por otra parte, las preguntas realizadas estaban orientadas en su mayoría a detectar ideación suicida y síntomas de depresión, dejando de lado un espectro mayor de resultados de salud mental orientados al bienestar psicológico. A partir de esto, a la fecha, se han introducido modificaciones en el cuestionario, con la intención de entregar una mirada más amplia a la salud mental. Por último, si bien Chile ha realizado importantes esfuerzos en salud mental (28), solo 2,16% del presupuesto nacional en salud se destina a esta área (29), por lo que la disponibilidad de dispositivos de atención de salud mental para toda la población residente en el país, tanto nacionales como extranjeros, se ve limitada.

Este estudio presenta algunas limitaciones a considerar. Por una parte, varias preguntas del cuestionario no fueron registradas de forma completa (por ejemplo, pertenencia étnica, forma de ingreso al país, con quien viajó, entre otras). Esto pudo deberse a la falta de tiempo en la atención o la extensión de dicho documento. Tal y como fue señalado, a la fecha se han realizado diversas ediciones al cuestionario para facilitar y acortar el proceso de registro durante la atención directa a personas migrantes internacionales. Otra limitación es que se interpretó como “demanda satisfecha” solo al cumplimiento efectivo de la atención de salud con el personal correcto (al que fue derivado) a un año de seguimiento. No fue objetivo de este estudio analizar el tiempo asociado al cumplimiento ni tampoco si la atención brindada por esta derivación fue considerada adecuada. No obstante, esta es un área de posible profundización futura y de revisión para la mejora permanente de este programa de atención inicial a migrantes internacionales en Chile.

## CONCLUSIONES

Desde el enfoque de equidad social en salud, resulta urgente buscar estrategias basadas en evidencia que acorten las brechas de acceso y uso efectivo de servicios de salud entre población migrante internacional y local en cualquier país del mundo. Una iniciativa concreta desarrollada y evaluada es el Programa de Atención Inicial a Migrantes desarrollado en Chile. A un año de seguimiento, esta intervención exitosa contó con alto nivel de satisfacción de parte de sus usuarios migrantes y se transformó en una puerta formal de entrada al sistema de salud público chileno. Este programa permitió, además, la detección de necesidades de salud específicas, como controles de embarazo y atenciones preventivas de salud, entre otros. Todas estas instancias de atención de salud tienen el potencial de proteger de mayor carga de enfermedad y costo futuro en este grupo y en el país en su conjunto. Los resultados obtenidos por este estudio pueden replicarse en todo el país, dado que la población migrante internacional atendida en la comuna de Santiago refleja la realidad migratoria que enfrentan otras comunas en Chile y, posiblemente, también otros países de la Región.

En el marco de la protección al derecho a la salud como un bien universal, se recomienda a todos los países de América Latina y el Caribe avanzar en la creación y adaptación de programas interdisciplinarios con enfoque de salud intercultural que permitan dar la bienvenida al sistema de salud a la población migrante internacional. El programa presentado puede servir de inspiración para dicho propósito.

## Contribución de las autoras.

MC colaboró en la concepción del estudio original, análisis e interpretación de datos, redacción de manuscrito. SA realizó el análisis e interpretación de datos y redacción de manuscrito. BC participó en la concepción del estudio original y colaboró en la redacción del manuscrito. Todas las autoras revisaron y aprobaron la versión final.

## Agradecimientos.

Las autoras agradecen a Paz Bersano, de la Ilustre Municipalidad de Santiago y a Margarita Bernales, de la Pontificia Universidad Católica de Chile.

## Financiamiento.

El modelo de atención presentado en el artículo fue realizado en el marco del Proyecto de Investigación FONDECYT 11130042 “Desarrollando inteligencia en salud pública primaria para migrantes internacionales en Chile: un estudio multi-métodos” (Comisión Nacional de Investigación Científica y Tecnológica, CONICYT, Chile).

## Declaración.

Las opiniones expresadas en este manuscrito son responsabilidad del autor y no reflejan necesariamente los criterios ni la política de la *RPSP/PAJPH* y/o de la OPS.
